# Fibrosis score 4 index has an independent relationship with coronary artery diseases in patients with metabolic-associated fatty liver disease

**DOI:** 10.1186/s13098-023-01031-y

**Published:** 2023-03-25

**Authors:** Maryam Namakchian, Soghra Rabizadeh, Sara Seifouri, Hassan Asadigandomani, Melika Arab Bafrani, Kiana Seifouri, Foroogh Alborzi Avanaki, Armin Rajab, Manouchehr Nakhjavani, Alireza Esteghamati

**Affiliations:** 1 Endocrinology and Metabolism Research Center (EMRC), Imam Khomeini Hospital Complex, Vali-Asr Hospital, Tehran University of Medical Sciences, Tehran, 13145-784 Iran; 2 Departments of Gastroenterology and Hepatology, Imam Khomeini Hospital Complex, Tehran University of Medical Sciences, Tehran, Iran

**Keywords:** Fatty liver disease, Type 2 diabetes, Coronary artery diseases, Fibrosis-4 (FIB-4) index

## Abstract

**Background:**

Metabolic-associated fatty liver disease (MAFLD), one of the most common liver diseases, is detected in patients with concomitant hepatic steatosis and Type 2 Diabetes (T2D). We looked into the relationship between Fibrosis-4 (FIB-4) index and coronary artery diseases (CAD) in patients with MAFLD, to further look into the efficiency of FIB-4 in screening for CAD among patients with MAFLD.

**Method:**

In this study, we included 1664 patients with MAFLD (T2D, who also had hepatic steatosis) during 2012–2022 and divided them into 2 groups; CAD and non-CAD. Demographic, Anthropometric indices, liver function tests, lipid profile and FIB-4 index of all patients were evaluated and compared.

**Result:**

Among the 1644 patients (all have MAFLD), 364(21.4%) had CAD. Patients with MAFLD and CAD were more probable to be hypertensive, have longer duration of diabetes and be older (with p-values < 0.001). After adjustment for confounding factors, in a multivariable logistic regression model, FIB4 showed a significant independent relationship with concomitant MAFLD and CAD. Upper Tertile FIB-4 had an odds ratio of 3.28 (P-value = 0.002) to predict CAD. Furthermore, in Receiver Operating Characteristic (ROC) Curve analysis with the maximum Youden Index, a FIB-4 cut-off of 0.85 (AUC = 0.656, 95% CI 0.618–0.693, P < 0.001) noted to predict CAD in patients with MAFLD.

**Conclusion:**

This study showed that the FIB-4 score independently correlates with CAD in patients with MAFLD.

## Introduction

The number of patients with diabetes is increasing dramatically around the world; it has been estimated that at the current rate there will be 634 million patients with diabetes in the world by 2030 [1].

One of the most common liver diseases worldwide is non-alcoholic fatty liver disease (NAFLD) which has become a public health problem in recent years [2]. Metabolic-associated fatty liver disease (MAFLD) is a novel terminology that was proposed recently by international experts instead of NAFLD in patients with overweight/obesity, type 2 diabetes, or evidence of metabolic dysregulation, in addition to hepatic steatosis [3]. MAFLD is important as it can further increase the risk of cardiovascular complications which can be fatal [4]. Various studies showed that by changing the definition of NAFLD to MAFLD, high percentage of people with fatty liver disease who had metabolic dysregulation may be in higher risk of developing coronary artery diseases (CAD) [5, 6].

The overall prevalence of MAFLD is about 39% among general population. Not only obese people, but lean and non-obese people are also vulnerable to MAFLD. It is worth noting that hypertension and diabetes are important comorbidities in non-obese patients with MAFLD [7, 8]. The main causes of death in MAFLD are cardiovascular, malignancy and end stage liver disease [9].

Liver biopsy is the gold standard for diagnosis of NAFLD. Because liver biopsy is an invasive procedure, alternative tools like FIB-4 which is non-invasive and inexpensive is used to estimate liver fibrosis in MAFLD [10]. FIB-4 index is calculated by using age, platelet count (PLT) and aspartate aminotransferase (AST) and alanine aminotransferase (ALT) levels. Hence, this index can be used for the early detection of liver fibrosis among patients with T2D [11]. The FIB-4 index is measured according the formula that follows: Age(years)* AST(Unit/Liter)/ (PLT (10^9^/L)*√ALT(Unit/Liter)) [12].

The aim of this study is to investigate the potential link between FIB-4 index and cardiovascular complications in patients with MAFLD.

## Study population

In this prospective study, patients with T2D referred to diabetes clinic of Vali-Asr hospital, affiliated with Tehran University of Medical Sciences during 2012 to 2022 were included. Patients with T2D based on the 2022 American Diabetes Association guideline [13] and non-alcoholic fatty liver disease based on ultrasound findings were included.

Those under the age of 18, with T1D, pregnancy, with a history of malignancy, end stage renal disease, heart failure or cirrhosis were excluded from the study. A total of 1644 patients with concomitant T2D and non-alcoholic fatty liver disease were included in this study, and then they were divided into two groups; patients with and without a coronary artery disease (CAD). In this study Patients with history of myocardial infarction, acute coronary syndrome [14], percutaneous coronary intervention (PCI), Coronary artery bypass graft (CABG) or angioplasty were considered to have CAD [15].

## Data collection

Patients’ baseline demographic and anthropometric characteristics including age, gender, duration of diabetes, history of hypertension (HTN), height, weight, and waist circumferences were recorded. Informed consent was obtained from all subjects according the declaration of Helsinki. All of the subjects were over 18 years old and all of them were qualified to give consent, so they filled the consent form individually.

Systolic and diastolic blood pressure, and laboratory data including fasting blood glucose (FBS), hemoglobin A1C (HbA1c), 2-hour post-prandial blood glucose (2hpp), creatinine, lipid profile including triglyceride (TG) ,cholesterol (Chol), low density lipoprotein cholesterol (LDL-C), high density lipoprotein cholesterol ( HDL-C), liver enzymes, insulin level, platelet count were measured. Urinary albumin excretion was measured using urinary albumin-to-creatinine ratio in random urine samples. Urinary albumin concentrations were evaluated by an immunoturbidimetric assay. Albuminuria was defined as the urine albumin-to-creatinine ratio greater than 30 mg/gr. Creatinine was measured by enzymatic method on automated analyzer. Homeostatic model assessment-Insulin Resistance (HOMA-IR) was calculated. Estimated GFR was calculated by the Modification of Diet in Renal Disease (MDRD) equation.

Waist circumferences were measured in upright position as the horizontal plane midway between the costal margins and the iliac crest. Hip circumference was measured as the distance around the largest part of the hip and Waist to Hip Ratio (WHR) was calculated by dividing waist circumference by hip circumference. For body mass index (BMI) calculation, weight (in kilograms) was divided by the square of height (in meters). Blood pressure was measured after 15 min of rest after patients arrived by using an automated blood pressure device. The mean of two blood pressure recordings, that were measured 10 min apart, was recorded. All blood samples were obtained after a 10–12 h of fasting and measured with kits certified by the central reference laboratory. HbA1c was recorded via high-performance liquid chromatography (A1C, DS5 Pink kit; Drew, Marseille, France). FBS was measured by enzymatic colorimetric methods with the glucose oxidase test and serum lipid profile (TG, HDL-C, LDL-C) were measured by enzymatic methods.

For the diagnosis of NAFLD based on imaging, at least two of the following three criteria were required: echogenic liver with an existing contrast compared with renal parenchyma, blurring of the vessels, and hepatic vein narrowing [16].

### Statistical analysis

All analyses were carried out using the 24th version of the SPSS software. P-values less than 0.05 were considered statistically significant. The normal distribution of the sample was tested with Kolmogorov-Smirnov and Shapiro-Wilk tests, p-p, plot and histogram. Continuous variables with normal distribution were expressed as means ± standard deviations (SD), and continuous variables with skewed distribution were expressed as median and interquartile range. T-test was conducted to differentiate these variables among patients with and without CAD and Mann Whitney U test was used for variables without normal distribution. Categorical variables were recorded as frequencies or proportions to evaluate the association of variables with CAD; chi-square analysis was applied where appropriate. Multivariate logistic regression analysis was performed to assess the relationship between FIB4 and other indicators with CAD. Odds ratios (ORs) that were calculated in the logistic regression analysis were expressed with a 95% confidence interval (CI). The area under the ROC (receiver operating characteristic) curve was estimated to determine the prognostic value of FIB4 for CAD in patients with MAFLD and the cut-off for FIB-4 was estimated using the Youden index.

## Results

A total of 1644 subjects with MAFLD were studied. These subjects were divided into two groups, including CAD and Non-CAD. The non-CAD control group consisted of 1280 (75.3%) patients, whereas the CAD group was composed of 364 (21.4%) patients.

Table 1 presents the baseline characteristics of the two groups. The mean age of CAD patients was 62.10 ± 10.182 and 61.5% (224) of them were male. The mean age of non-CAD controls was 53.02 ± 11.023 and 48.5% (621) of them were male. According to Table 1, participants with MAFLD and CAD were more likely to be older, male, hypertensive, had longer duration of diabetes, and increased frequency of albuminuria compared to patients without CAD (with all P-values < 0.001). Also, their FBS and 2hpp (2-hour post-prandial) levels were higher (with all P-values < 0.001). These patients were shown to have higher levels of SBP (P-value = 0.017) and HbA1C (P-value = 0.017), waist to hip ratio (P-value = 0.015) compared to the non-CAD patients. Whereas, the opposite was true for the levels of AST (P-value = 0.010), ALT (P-value = 0.002), ALKP (P-value = 0.005), and eGFR (P-value < 0.001). They were shown to have lower levels of cholesterol, triglyceride and LDL than their non-CAD counterparts.

**Table 1:** Comparison of baseline characteristics of patients with MAFLD with and without CAD

	Non-CAD N = 1280	CAD N = 364	P-Value
**Age, years**	53.02 ± 11.02	62.10 ± 10.18	**< 0.001**
**Duration of DM, years**	8.87 ± 6.69	14.97 ± 8.79	**< 0.001**
**Gender (female/male)**	51.5% (659) /48.5% (621)	38.5% (140) / 61.5% (224)	**< 0.001**
**Waist circumference, cm**	103.36 ± 11.09	103.72 ± 10.02	0.55
**Waist/Hip**	0.94 ± 0.05	0.95 ± 0.05	**0.01**
**SBP, mmHg**	128.62 ± 33.68	133.00 ± 16.98	**0.017**
**DBP, mmHg**	79.75 ± 7.78	79.68 ± 8.81	0.90
**HTN**	34.7% (443)	58.5% (213)	**< 0.001**
**BMI, kg/m²**	31.36 ± 5.63	31.06 ± 5.14	0.37
**platelet**	275 ± 52.7	260 ± 59.80	**< 0.001**
**FBS, mg/dl**	155.55 ± 52.25	169.82 ± 67.06	**< 0.001**
**2hpp, mg/dl**	211.98 ± 83.58	231.28 ± 83.71	**< 0.001**
**Hb AIC, %**	7.591 ± 1.61	7.80 ± 1.45	**0.01**
**Cholesterol, mg/dl**	186.20 ± 43.95	168.33 ± 43.46	**< 0.001**
**HDL-C, mg/dl**	44.40 ± 11.73	43.92 ± 11.16	0.48
**LDL-C, mg/dl**	105.91 ± 34.22	91.32 ± 32.86	**< 0.001**
**TG, mg/dl**	195.86 ± 141.84	169.39 ± 83.77	**< 0.001**
**Creatinine**	0.97 ± 0.23	1.04 ± 0.23	**< 0.001**
**AST, U/L**	29.29 ± 17.83	26.85 ± 14.92	**0.010**
**ALT, U/L**	41.07 ± 23.43	36.58 ± 24.29	**0.002**
**ALKP, U/L**	169.77 ± 88.35	153.34 ± 72.30	**0.005**
**eGFR,mL/min/1.73 m²**	103.96 ± 31.62	95.98 ± 27.25	**< 0.001**
**Albuminuria**	17.3% (134)	28.4% (74)	**< 0.001**
**HOMA-IR**	4.05 (2.72–5.87)	4 (2.80–5.65)	0.44
**Smoking % (n)**	4.6% (59)	6% (21)	0.43
**FIB4**	0.94 ± 0.47	1.15 ± 0.48	**< 0.001**
**Insulin levels**	12.57 ± 7.53	11.36 ± 5.97	**0.003**
**Statin use** **Antidiabetic drugs**	56.2%	74.1%	**< 0.001**
**Oral agents**	84.3%	84.1%	0.50
**Oral agents + Insulin**	12.7%	11.6%	
**Insulin**	2.9%	4.3%	

Data are presented as mean ± standard deviation, median (interquartile range), or counts (percentages).MAFLD: metabolic-associated fatty liver disease, DM: diabetes mellitus, SBP: systolic blood pressure, DBP: diastolic blood pressure, BMI: body mass index, FBS: fasting blood glucose, HBA1C: hemoglobin A1C, HDL-C: high-density lipoprotein cholesterol, LDL-C: low-density lipoprotein cholesterol, TG: triglyceride, AST: aspartate transaminase, ALT: alanine transaminase, ALP: alkaline phosphatase, eGFR: estimated Glomerular Filtration Rate, HOMA-IR: homeostatic model assessment of insulin resistance

BMI (P-value = 0.372), waist circumference (P-value = 0.556), smoking (P-value = 0.434), HDL-C (P-value = 0.489), HOMA-IR index (P-value = 0.397) and, DBP (P-value = 0.902), did not differ between MAFLD patients with and without CAD.

Nonetheless, FIB4 index was significantly higher among patients with both MAFLD and CAD compared those without CAD (1.15 ± 0.48 versus 0.94 ± 0.47) respectively (P-value < 0.001).

As shown in Table 2, we divided subjects according FIB-4 tertile scores to shows the number and proportion of patients with MAFLD and CAD in each group. While the lower tertile consists of 29 (12.6%) patients with MAFLD and CAD, the middle and upper tertile, include 77 (33.3%) and 125 (54.1%), respectively.

**Table 2:** Prevalence of CAD in three groups according to FIB-4 tertile in patients with MAFLD

FIB-4 tertile	Non-CAD	CAD	P-value
Tertile 1st (0.22–0.74)	272 34.1%	29 12.6%	< 0.001
Tertile 2nd (0.746–1.062)	243 30.5%	77 33.3%	< 0.001
Tertile 3rd (1.063–3.78)	283 35.5%	125 54.1%	< 0.001

*FIB-4* fibrosis score index, *CAD* coronary artery disease (the percentage of patients with CAD in the second and third tertiles of fib-4 is significantly higher compared to first tertile. While this percentage is significantly higher in Non-CAD patients in the first tertile of fib-4, these results confirm that higher fib-4 is associated with CAD).

In multivariable logistic regression analysis, FIB4 index had a significant relation with CAD in those with MAFLD. This relationship was remained significant after adjusting for multiple confounding factors including gender, age, smoking, duration of diabetes, BMI, waist-to-hip ratio, HTN, HbA1c, HDL-C, LDL-C, TG and eGFR. The odd’s ratio for the middle tertile of FIB4 index compared to lower tertile showed to be 2.59 with a P-value = 0.008, and the upper tertile had a higher odd’s ratio, at about 3.28 and a P-value = 0.002. (Table 3)

**Table 3:** Results of Multivariate logistic regression analysis

	Beta	Standard error	Odd’s ratio	95% C.I.		P-value
				Lower	Upper	
FIB-4 index: (Reference)Lower tertile	-	-	-	-	-	0.004
Middle tertile	0.953	0.361	2.594	1.277	5.268	0.008
Upper tertile	1.188	0.360	3.280	1.621	6.638	0.001
**Gender (male)**	1.011	0.308	2.749	1.504	5.026	0.001
**Age**	0.028	0.016	1.028	0.996	1.062	0.087
**Duration of DM**	0.094	0.017	1.098	1.063	1.135	0.000
**History of HTN**	0.167	0.232	1.181	0.750	1.860	0.472
**Smoking**	1.070	0.661	2.915	0.797	10.657	0.106
**LDL-C**	-0.015	0.004	0.985	0.978	0.992	0.000
**TG, mg/dl**	0.001	0.001	1.001	0.998	1.003	0.468
**Hb A1c**	-0.019	0.078	0.981	0.841	1.144	0.807
**eGFR**	-0.016	0.007	0.984	0.971	0.997	0.016
**Albuminuria**	0.287	0.275	1.332	0.778	2.281	0.297

FIB-4: Fibrosis score 4, FIB-4 first tertile is considered as reference, WHR: waist to hip ratio, eGFR: estimated glomerular filtration rate measured in mL/min/1.73 m², BMI: body mass index, DM: diabetes mellitus, HTN: hypertension, HDL-C: high density lipoprotein cholesterol, TG: triglyceride, HBA1c: hemoglobin A1C

**Fig. 1:**
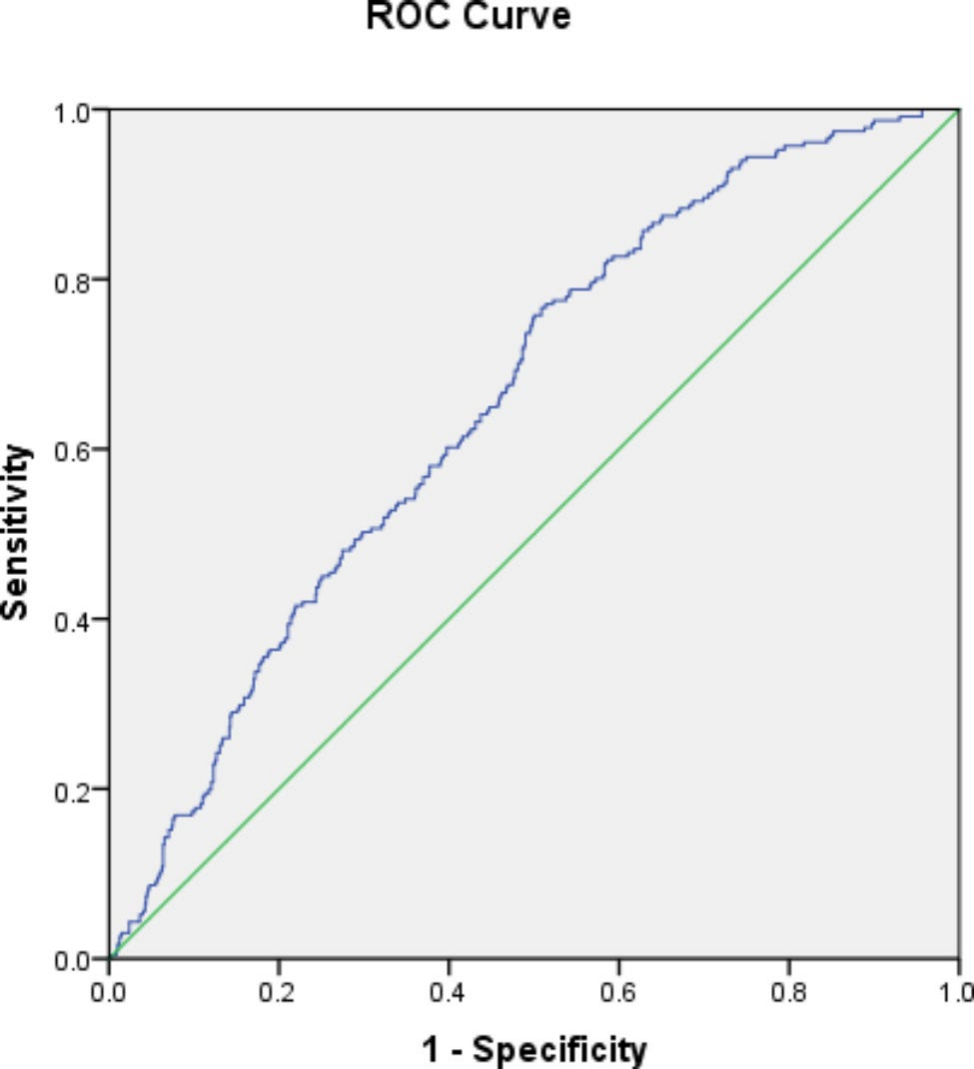
AUROC curve for FIB-4

In ROC analysis the predictive value of FIB4 index for CAD in patients with MAFLD is illustrated in Fig. 1; Table 4. With the maximum Youden Index, the cut-off was set at 0.85 with a sensitivity of 75% and specificity of 50%. (AUC = 0.656, 95% CI 0.618–0.693, P < 0.001).

**Table 4:** Multivariate logistic regression analysis

	AUC	95% CI	Sensitivity	Specificity	Cut-off
**FIB-4**	0. 656	0.618–0.693	75%	50%	0.85

*FIB-4* Fibrosis-4 index, *AUC* area under the curve, *AUROC* area under the receiver operating characteristic

## Discussion

In this study, the relationship between FIB-4 index and coronary artery disease in patients with MAFLD was evaluated. The results of the present study showed that after adjustment for multiple confounding factors, patients with a higher FIB-4 score are 2.5 to 3 times more probable to have CAD. The FIB-4 index had an independent relationship with CAD in a multivariable logistic regression.

A new definition of metabolic-associated fatty liver disease or “MAFLD” reflects metabolic dysregulation much better than NAFLD, because the term of NAFLD emphasizes just on “non-alcoholic” but MAFLD insinuates metabolic causes of liver disease. These criteria of MAFLD are based on the presence of hepatic steatosis in the presence of one or more of overweight/obesity, type 2 diabetes mellitus, or evidence of metabolic dysregulation [17]. Due to increasing frequency of obesity, and diabetes we are facing with a surge of metabolic disorders in children and adolescents. The criterion for a diagnosis of pediatric MAFLD is based on liver histopathology or imaging, serum biomarkers or score of hepatic steatosis with at least one of these criteria: excess adiposity, T2DM, or any evidence of metabolic dysregulation [18].

Certain genetic causes are involved in the etiology of MAFLD. Studies have shown that the breakdown of toll-like receptor (TLR) tolerance can lead to tissue damage and the activation of TLR causes inappropriate inflammatory reactions that have been implicated in the severity of MAFLD [19]. Studies has now focused on genome-wide association studies (GWAS) to discover via multi-trait GWAS, genome-wide association studies (PheWAS), Mendelian randomization and functional annotation studies [20].

Liver fibrosis is associated with CAD risk factors such as obesity, hypertension, diabetes, dyslipidemia, etc. NAFLD can impact the severity of atherosclerosis [21]. In multiple studies, the relation between NAFLD and CAD has been detected, indicating that patients with NAFLD have a higher chance of developing CAD and its life-threatening complications [22–24]. In contrast, a study by Ken Liu, et al. in 2017, reported that the amount of fat in the liver, as measured by controlled attenuation parameter (CAP), did not correlate with the incidence of cardiovascular events [25].

Though the definite cause behind the increased rate of CAD among patients with hepatic steatosis has not been determined as of yet, some speculations have been made. One of which is that NAFLD is commonly associated with T2D, a comorbidity also known as MAFLD, leading to insulin resistance and increased blood glucose. These all can in turn further increase the chances of patients developing CAD by triggering monocyte/macrophage adhesion to the vascular walls, and stimulating chemokine secretion by the smooth muscle cells of the vessels, and activating inflammation via macrophages [26]. In line with the findings in patients with NAFLD, MAFLD can potentially influence the risk of CAD. This is because of the overlap between NAFLD and MAFLD as well as the more metabolic derangements in patients with MAFLD, which in turn further increases the risk of CAD [27].

Many specialists refer patients that are at intermediate or high-risk of developing CAD to cardiologists for a cardiovascular review. That being said, currently no screening tool has been proven to be efficient enough to be adopted for asymptomatic patients with MAFLD [28].

In 2019, Song et al. carried out a study on patients with NAFLD without CAD and concluded that FIB-4 score as a noninvasive fibrosis marker is significantly associated with the coronary artery calcium score (CACS) > 100 [29]. Also Lee, J. et al. showed the association between intermediate/high FIB-4 scores and the progression of coronary artery calcification (CAC) in patients with NAFLD [30].

Tsai, T.Y, et al. study in 2022 revealed that patients with atherogenic plaque in the coronary computed tomography angiography had higher FIB4 and other liver fibrosis scores including Forns score, and NFS [31].

Jin, J.L, et al. study showed that in patients with established CAD in the general population, the FIB4 index had a positive relationship with the number of diseased vessels [32] .

In 2022 Chen, X., et al. stated that the FIB-4 and other noninvasive liver fibrosis scoring systems (NFS, APRI, and BARD) are useful in assessing advanced fibrosis for patients with MAFLD [33] .

In the CORONASH study, carried out in 2021, association of FIB4 index with advanced liver fibrosis in patients with established CAD was evaluated. One hundred eighty nine patients with proven CAD were assessed for a concomitant advanced liver fibrosis disease with the use of 5 different non-invasive fibrosis tests. This study showed that about 5% of patients with established CAD had advanced liver fibrosis. They propose the use of non-invasive fibrosis tests in CAD patients to avoid non necessary further assessment (e.g. by Fibro scan, then liver biopsy) [34]. In our study, we mainly focused on the relationship between FIB-4 levels in patients with concomitant MAFLD and CAD. To do this, the predictive value of FIB-4 was measured after adjusting for multiple confounding factors including Age, Gender, Duration of DM, HTN, Smoking, BMI, GFR, Waist/Hip ratio, HbA1c, lipid profile, and albuminuria. Our findings were in line with the CORONASH study with regards to AUROC of FIB-4, here it was estimated at approximately 0.656, which was slightly higher compared to that of the CORONASH study (0.647).

In a prospective cohort study Chen, Q., et al. followed 3263 patients with established CAD in general population with regards to their mortality rate. 319 deaths were identified due to cardiovascular diseases. They showed that patients with the highest FIB-4 score levels had more cardiovascular mortality compared to those with the lowest FIB-4 score [35].

Han, E., et al. calculated the ASCVD risk scores among general population, where the prevalence of MAFLD was 38.0%. They concluded that in patients with MAFLD, higher FIB-4 score correlates with higher ASCVD risk score [27]. According to high global prevalence of obesity and other associated disease including diabetes, metabolic dysfunction-associated fatty liver disease (MAFLD), hypertension, CAD, malignancy and HTN a primary care-driven, patient-centered, multidisciplinary model is needed to provide a holistic care with focus on clinical care and new clinical trials study for management of metabolic diseases [36].

In the present study, a significantly lower LDL-C, TG and cholesterol levels were observed in the CAD group compared to the non-CAD group. We think that it may be due to suggested healthy diet, health conscious lifestyle, and statin use in patients with both MAFLD and CAD, that were recorded in the patients files.

The present study showed that FIB-4 score with a cut-off of 0.85 and AUROC of 0.656 may potentially play a role in prediction of CAD in patients with MAFLD.

However, we suggest further investigations should be conducted on the correlation between FIB-4 and CAD in patients with MAFLD.

## Conclusion

This study showed the relationship between FIB-4 index and coronary artery disease in patients with MAFLD. Hence, due to increased global prevalence of MAFLD, we suggest that this simple and non-invasive index to be investigated in further studies.

## Data Availability

The data that support the findings of this study are available on request from the corresponding author.
